# Biophysical Induction of Vascular Smooth Muscle Cell Podosomes

**DOI:** 10.1371/journal.pone.0119008

**Published:** 2015-03-18

**Authors:** Na Young Kim, Julie C. Kohn, John Huynh, Shawn P. Carey, Brooke N. Mason, Ageliki G. Vouyouka, Cynthia A. Reinhart-King

**Affiliations:** 1 Department of Biomedical Engineering, Cornell University, Ithaca, New York, United States of America; 2 Divison of Vascular Surgery at Mount Sinai Hospital, New York, New York, United States of America; Tel Aviv University, ISRAEL

## Abstract

Vascular smooth muscle cell (VSMC) migration and matrix degradation occurs with intimal hyperplasia associated with atherosclerosis, vascular injury, and restenosis. One proposed mechanism by which VSMCs degrade matrix is through the use of podosomes, transient actin-based structures that are thought to play a role in extracellular matrix degradation by creating localized sites of matrix metalloproteinase (MMP) secretion. To date, podosomes in VSMCs have largely been studied by stimulating cells with phorbol esters, such as phorbol 12,13-dibutyrate (PDBu), however little is known about the physiological cues that drive podosome formation. We present the first evidence that physiological, physical stimuli mimicking cues present within the microenvironment of diseased arteries can induce podosome formation in VSMCs. Both microtopographical cues and imposed pressure mimicking stage II hypertension induce podosome formation in A7R5 rat aortic smooth muscle cells. Moreover, wounding using a scratch assay induces podosomes at the leading edge of VSMCs. Notably the effect of each of these biophysical stimuli on podosome stimulation can be inhibited using a Src inhibitor. Together, these data indicate that physical cues can induce podosome formation in VSMCs.

## Introduction

Podosomes are dynamic actin-rich cellular structures capable of adhering to and degrading extracellular matrix (ECM) [1–3]. As such, podosomes are largely found in invasive cell types, including macrophages, osteoclasts, and dendritic cells [4,5]. Podosomes are also found in vascular smooth muscle cells (VSMCs) and are thought to mediate VSMC migration [1,6,7,8] and may contribute to intimal hyperplasia in response to vascular injury or atherosclerosis. More recently, VSMCs have been shown to form podosomes when induced by phorbol esters [6] or growth factors [7]. Although the release of growth factors due to inflammation or other pathological conditions has been regarded a critical factor inducing podosome formation [7], very little is known about the physical cues in the vascular microenvironment that induce and mediate podosome formation.

During intimal hyperplasia, VSMCs convert from a contractile, quiescent phenotype to a synthetic, invasive phenotype and migrate from the media to the intima [9]. Many physiological factors are implicated in the initiation of VSMC migration, such as growth factor stimulation [9,10], topography [11,12], pressure [13], and injury [14]. However, podosome formation in VSMCs are typically studied solely in response to phorbol esters or growth factors, which do not completely mimic the conditions within the native or diseased microenvironment. Since VSMCs are capable of sensing and responding to force and physical cues, we hypothesized that VSMC podosomes may be induced by these same stimuli.

Using engineered platforms to recreate aspects of the microenvironment of the blood vessel wall, we show that topographical cues, static pressure, and injury can trigger podosome formation. Inhibition of Src or cdc42 pathways suppresses the formation of podosomes formed in response to these physical cues. Although our data is specific to VSMCs, these novel mechanisms of podosome formation could be applicable to other invasive cell types.

## Materials and Methods

### Cell culture

A7R5 rat aortic smooth muscle cells (ATCC, Manassas, VA) were maintained in DMEM (Invitrogen, Carlsbad, CA) supplemented with 10% fetal bovine serum (Invitrogen) and 100 units/ml of penicillin/streptomycin (Invitrogen) at 37°C in a 5% CO_2_ incubator. A7R5 were chosen as they have been widely used in the literature and closely recapitulate podosomes found in humans [8, 15, 16]. In inhibitor studies, cells were incubated in 10 μM 4-amino-5-(4-methylphenyl)-7-(t-butyl)pyrazolo-d-3,4-pyrimidine (PP1) (Sigma-Aldrich, St. Louis, MO) for 30 min before further experimentation [17,18]. In transfection studies, cells were transfected with 1 μg/ml actin-eGFP (a gift from David Holowka, Department of Chemistry and Chemical Biology, Cornell University, Ithaca, NY) or 0.5 μg/ml actin-eGFP and 0.5 μg/ml cdc42T17N (a gift from Richard A. Cerione, Department of Molecular Medicine, Cornell University, Ithaca, NY) in 3 μg/ml Lipofectamine 2000 (Invitrogen). Media was replenished after 4 hours of lipofection.

### Immunofluorescent staining

Cells were fixed with 3.7% formaldehyde in PBS and permeabilized with 1% Triton-X. Cells were then immunostained with rabbit polyclonal cortactin primary antibody (H-191, Santa Cruz Biotechnology, Santa Cruz, CA) and Alexa Fluor 594 goat anti-rabbit secondary antibody (Invitrogen) or phospho-Src (Tyr416) Antibody (2101, Cell Signaling Technology, Danvers, MA) and Alexa Fluor 488 donkey anti-rabbit secondary antibody (Invitrogen). F-actin was stained with Alexa Fluor 488 conjugated phalloidin (Invitrogen), and nuclei were stained with 4′,6-diamidino-2-phenylindole (DAPI) (Sigma-Aldrich). Images were taken on a LSM 700 confocal microscope (Zeiss, Germany) or an Axio Observer.Z1m microscope (Zeiss, Germany). The presence of podosomes was determined by co-localization (yellow) of F-actin (green) with cortactin (red), a common marker of podosomes [8].

### Patterned polyacrylamide gel synthesis

Patterned 30 kPa polyacrylamide gels (PA) were prepared as previously described [19]. Briefly, 6%:0.28% acrylamide:bis-acrylamide (Bio-Rad, Hercules, CA) was polymerized between a patterned silanized silicon mold and an activated 22 x 22 μm^2^ coverglass. The pattern features were 10 × 10 × 200 μm (W × H × L). Following the release of gels from the mold, gels were with 0.1 mg/ml rat tail collagen type I (BD Biosciences). Cells were seeded onto the patterned gels on the coverslips in 6 well plates and incubated for 24 hr before fixing and subsequent immunostaining. Only the cell regions attached to the horizontal plane of the PA gels were observed since significant reflectance along the vertical edges of the wall made podosome observation difficult.

### Wound healing assay

Actin-eGFP transfected cells were wounded by dragging a sterile 200 μl pipette tip across confluent monolayers to create cell-free zones. Wounded monolayers were then observed for 12 hr using fluorescence, time-lapse microscopy.

### Pressure Assay

A custom-built aluminum pressure chamber [20] was placed in a 37°C incubator. The oxygen level was controlled at an ambient level of 21% for both the pressure chamber and the incubator. A gas tank (95% air, 5% CO2, Airgas, Salem, NH) was used for the experiment. For pressurized conditions, the compressed air and CO_2_ were delivered by two regulators in series to achieve an environment with desired pressure levels and 5% CO_2_. Cells seeded in 6-well plates were placed inside the chamber and pressurized to 100, 150, or 200 mmHg for 30 minutes. Cells were fixed and stained for cortactin, actin, and nuclei. For pSrc studies, cells were stained for actin and pSrc f (Tyr416) (Cell Signaling, Danvers, MA). Cells were imaged using a Zeiss LSM700 inverted laser scanning confocal microscope (Carl Zeiss, Oberkochen, Germany).

### Western blotting

Following 30 min of exposure to 200 mmHg, cells were immediately lysed using preheated (95°C) 2× Laemmli sample buffer. Protein extract was subjected to sodium dodecyl sulfate (SDS)-polyacrylamide gel electrophoresis, electro-transferred onto a polyvinylidene difluoride membrane and the blots were incubated with primary antibody overnight at 4°C followed by horseradish peroxidase-conjugated secondary antibody for 1 hour at room temperature. Primary antibodies used were anti-pSrc (Tyr416) (Cell Signaling, Danvers, MA) and anti-GAPDH (Millipore, Billerica, MA). Signal was detected using the SuperSignal West Femto kit (Thermo Scientific, Rockford, IL). For quantification, p-Src signal was normalized to GAPDH. Data are presented as mean ± SE for three independent experiments.

### Statistical analysis

All experiments were performed at least in duplicate and data are reported as mean ± SD. All data were tested for normality using the Shapiro-Wilk goodness-of-fit test on JMP software. To compare two groups of data, statistical significance was determined by Student’s t-test for normal data and with the Wilcoxon rank sum test for non-normal data. For multiple comparison tests where residual distributions failed normality tests, the Kruskal-Wallis one-way analysis of variance with Dunn’s multiple comparison test was used to determine statistical significance. Both GraphPad Prism (v. 5.0) and JMP Pro (v. 11) software were used and P values < 0.05 were considered statistically significant.

## Results

### Podosome formation is induced by topography

**Fig 1:**
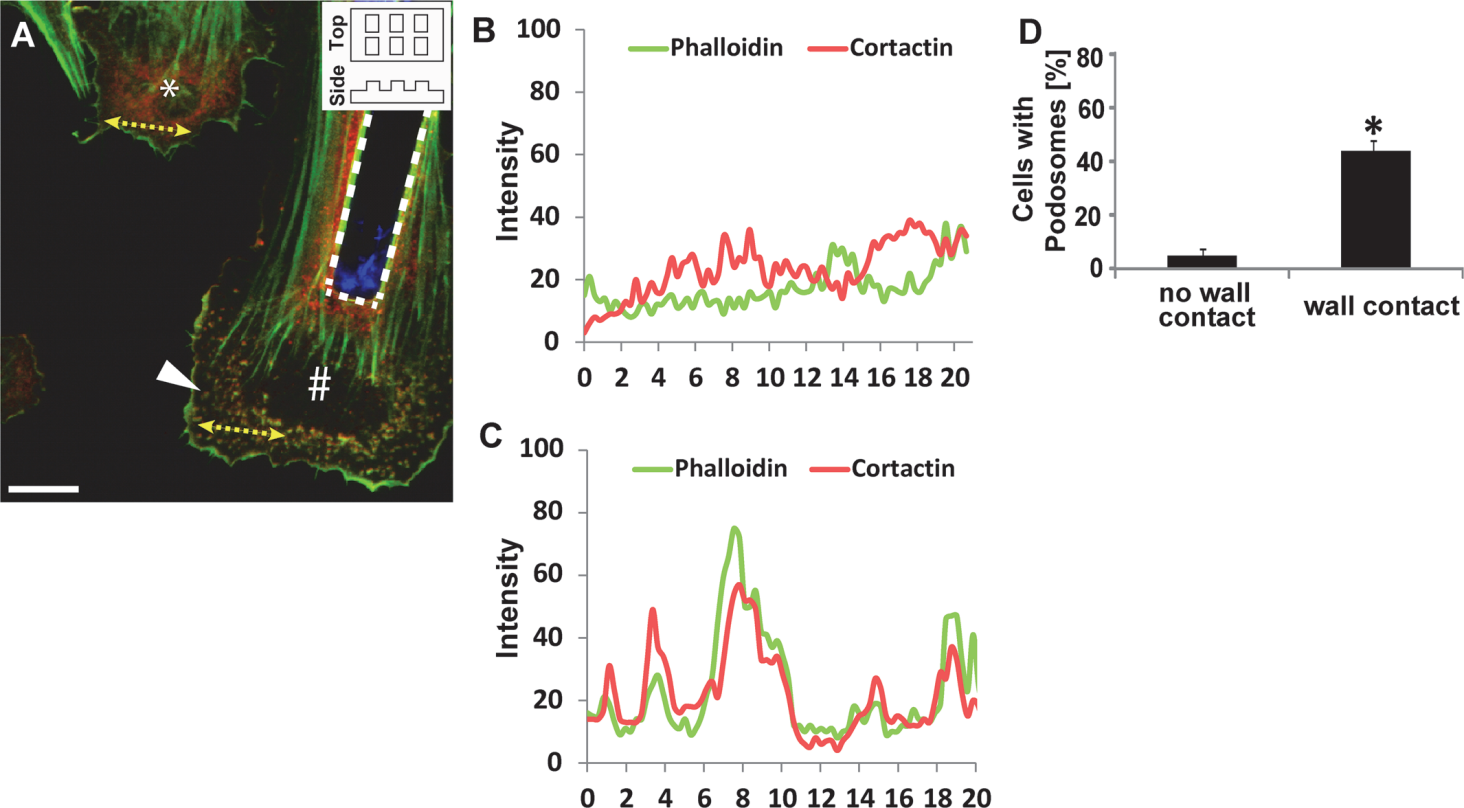
Physical barriers induce formation of podosomes. (A) Representative confocal image of A7R5 aortic smooth muscle cells seeded on a micromolded 30 kPa collagen-coated polyacrylamide (PA) gel containing a vertical wall. A schematic of the top and side views of the molded PA gel are illustrated in the inset in the upper right corner. Podosomes were identified based on the colocalization of actin (green) and cortactin (red). Nuclei (blue) were stained with DAPI. The cell denoted by the asterisk (*) is not in contact with a wall whereas the cell denoted by the crosshatch (#) is adhered to a wall (dotted white line). The white arrowhead indicates punctate podosome formation. Scale bar, 30 μm. (B and C) Intensity profiles of actin and cortactin staining of the yellow dotted line (20 μm) (depicted in A) drawn in the cell without wall contact (B) and with wall contact (C). (D) Percentage of cells exhibiting podosomes when in contact or not in contact with a wall. Data are mean ± S.D. * P < 0.05 (Student’s *t*-test, n = 4).

Several mechanical and structural cues exist in diseased arteries due to matrix rearrangements that occur during disease progression and in treated arteries due to stent geometry [21]. As both cell adhesion and migration have been shown to be affected by topographical cues [22,23], we hypothesized that cell contact with a physical barrier, analogous to VSMCs contacting a stent *in vivo*, may induce podosome formation. To create three-dimensional substrate features, cells were seeded on 30 kPa polyacrylamide (PA) gels patterned with 10 μm tall vertical walls. Cells were considered “in contact” with a wall when any portion of the cell body was in contact with a wall, regardless of contact area. When cells were not in contact with micropost walls, cortactin, a widely used podosome marker [8], was diffuse throughout the cells (Fig. 1A, left), exhibiting no colocalization with actin. However, when cells contacted micropost walls (Fig. 1A, right), cortactin co-localized with actin in punctate spots, indicating the presence of podosomes. To more rigorously identify podosomes, a line segment was drawn across the cell edge (Fig. 1A, dotted line) and the intensity of red and green fluorescence was quantified (Figs. 1B and C). Overlapping curves (Fig. 1C) indicate the presence of a podosome. Podosomes were found primarily at the cell edge when contacting the wall. It is interesting to note that no podosomes were observed along the top horizontal surface of the wall. The average number of cells that exhibit podosomes was quantified and compared between the conditions in which cells were and were not in contact with micropost walls (Fig. 1D). We found an approximate 9-fold increase in the number of cells with podosomes that were in contact with a vertical wall as compared to those not in contact with a wall.

### Podosomes are induced in response to injury

**Fig 2:**
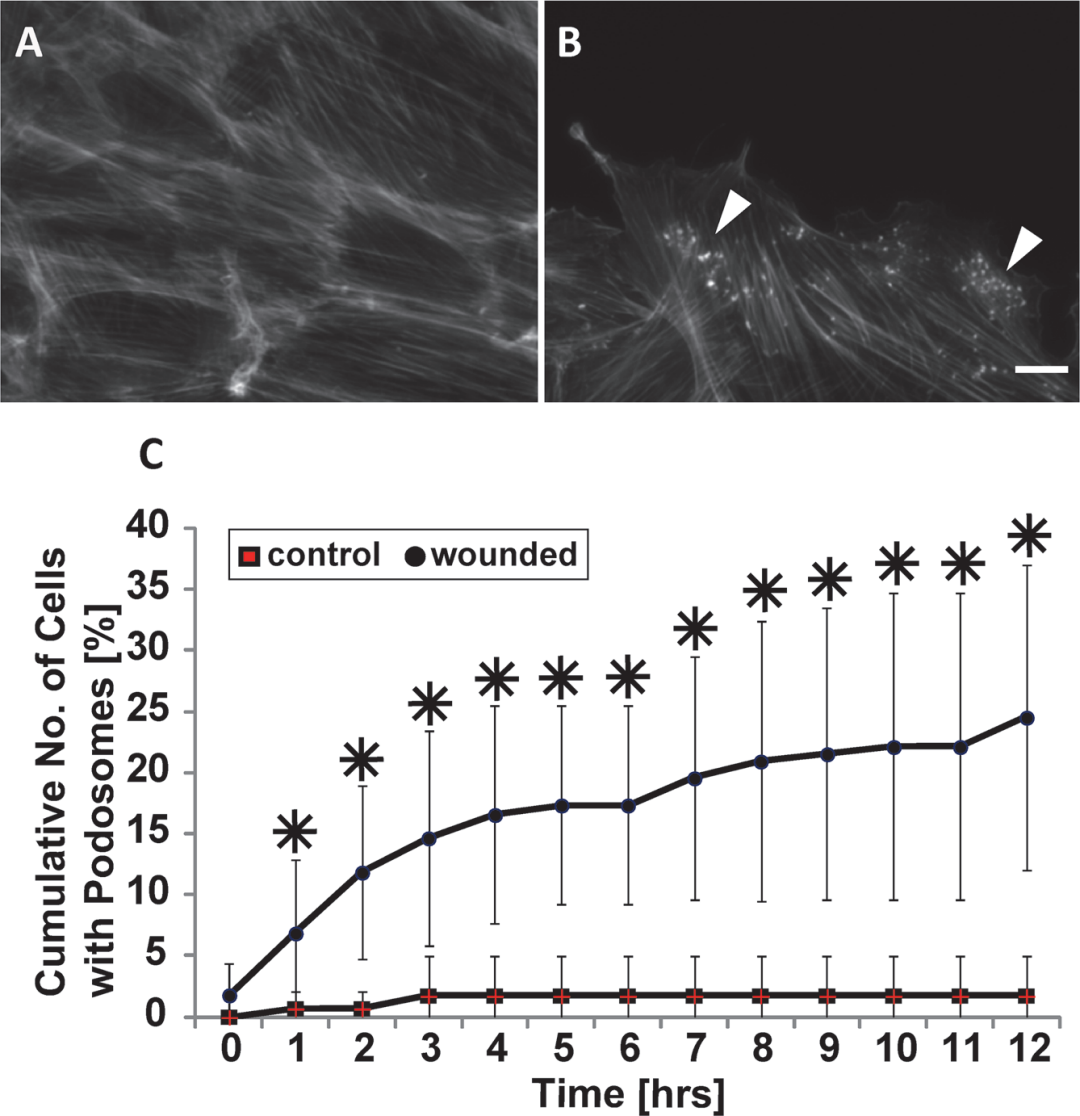
Podosomes form in response to wounding. (A and B) Representative images of confluent cells stained for actin without (A) or with wounding (B). White arrowheads indicate podosomes. Scale bar, 20 μm. (C) Quantification of the cumulative number of cells exhibiting podosomes with respect to time. Data are mean ± S.D. * P < 0.05 (Wilcoxon rank sum test, n = 3).

Given our finding that the physical cue of contact with a wall contact promotes podosome formation (Fig. 1), we investigated whether other contact cues also stimulate podosomes. It is known that VSMCs in the medial layer of arterial walls undergo a phenotypic modulation in response to injury to the vessel wall [8] which can stimulate VSMC migration. As such, we hypothesized that wounding of a VSMC monolayer, which induces migration, may promote podosome formation. To test our hypothesis, we performed a scratch assay on confluent monolayers of VSMCs. While unwounded confluent VSMCs rarely formed podosomes (Fig. 2A), cells at and near the wound continuously formed dynamic podosomes until wound closure occurred (Fig. 2B). The cumulative number of cells with podosomes within five cell rows from the wound edge was quantified and plotted as a function of time (Fig. 2C). As a control, we counted the number of cells exhibiting podosomes along a straight line within an unwounded dish. Throughout the wound healing process, the cumulative number of cells exhibiting podosomes significantly increased compared to control.

### Podosome formation is induced by increased static pressure

**Fig 3:**
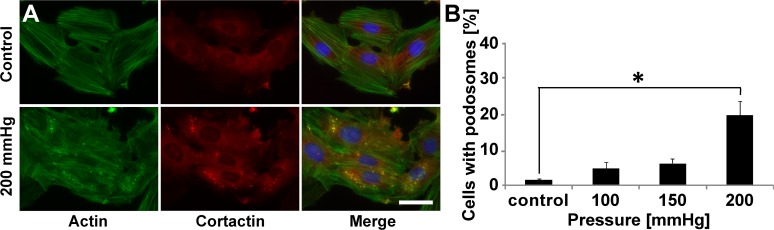
Hypertensive pressure induces podosome formation. (A) Representative images of A7R5 cells cultured without pressure (control) or with 200 mmHg (mimicking hypertension). Scale bar, 50 μm. Merge includes DAPI. (B) Percentage of cells exhibiting podosomes as a function of pressure. Data are mean ± S.D. * P < 0.05 (Kruskal-Wallis one-way analysis of variance with Dunn’s multiple comparison test).

Given our finding that VSMCs respond to topographical cues by increasing podosome formation, we sought to investigate whether other physical cues within the diseased vascular microenvironment known to induce migration also induce podosomes in VSMCs. Evidence indicates hypertensive pressures can alter VSMC actin polymerization [24], and because of the key role of actin in podosome structure [2,5,25], we examined the effect of elevated static pressure on podosome formation. Cells were stimulated with 100, 150, and 200 mmHg of pressure, which mimics physiological, stage I hypertension, and stage II hypertension [26] pressures, respectively. Non-pressurized cells served as a control. Notably, pressure induces podosome formation in VSMCs (Fig. 3A). Moreover, podosome induction by pressure is dependent on the magnitude of the pressure that is applied (Fig. 3B). Higher pressures, mimicking those found in hypertensive patients cause increased podosome formation.

### Pressure induces Src phosphorylation

**Fig 4:**
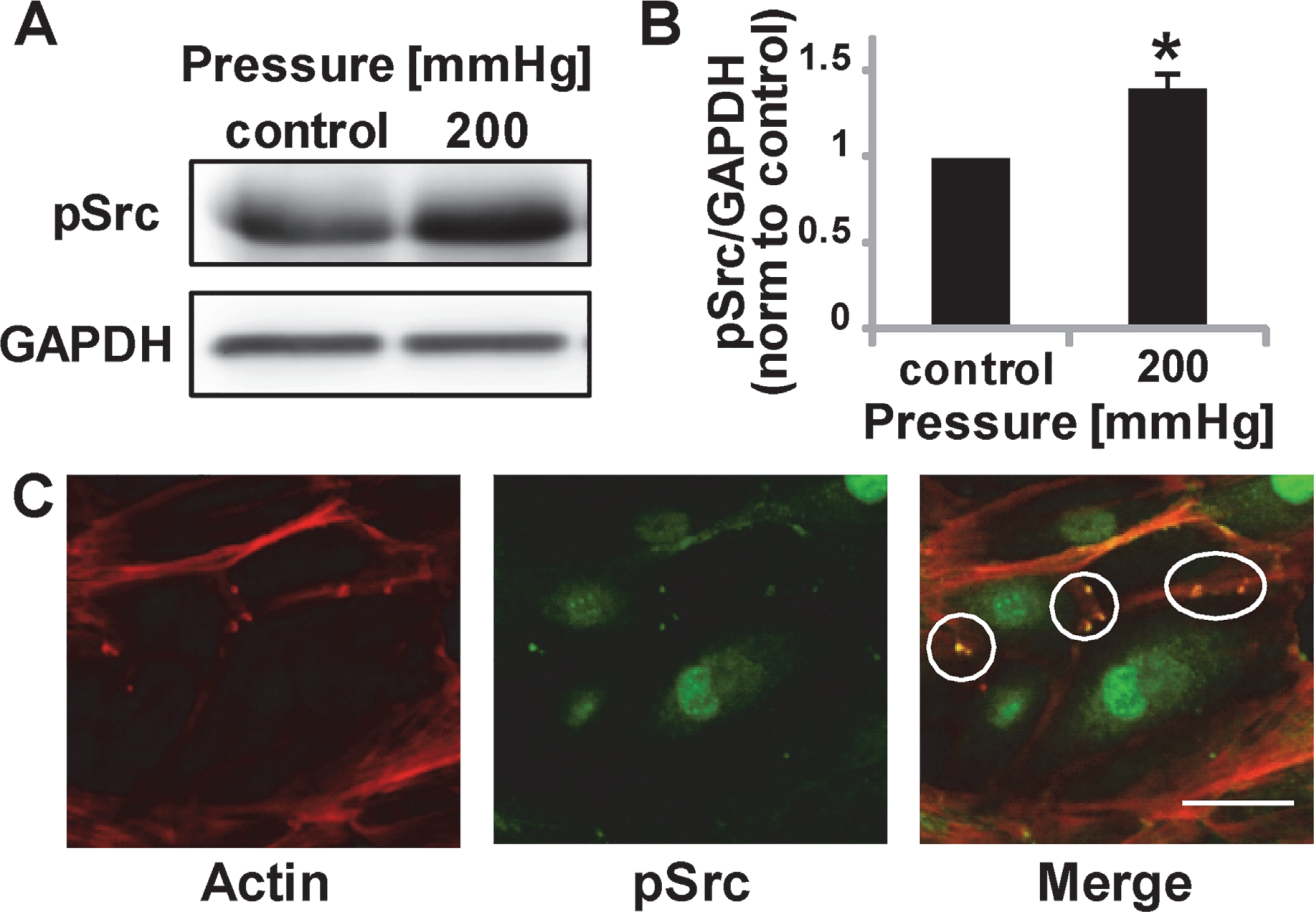
Phosphorylated Src is upregulated by a physiological pressure stimulus. (A) Representative Western Blot image of pSrc and loading control. (B) pSrc quantification after 30 minutes of pressure stimulation, normalized to control conditions. Data are mean ± S.D.* P < 0.05 (Student’s *t*-test, n = 5 from two independent experiments) (C) pSrc and podosomes show co-localization, circles indicate podosome regions. Scale bar, 50 μm.

Our study suggests that topography, wounding, and increased pressure induce podosomes. However, it is not clear how these physical cues are mechanotransduced to promote podosome formation. Evidence suggests Src kinase activity is sufficient to induce podosome formation, as shown by the ability of v-Src-transformed fibroblasts, which exhibit constitutively active Src, to form podosomes [27]. As such, we assayed for Src phosphorylation in cells subjected to 200mmHg pressure. Notably, pressure induces Src phosphorylation (Fig. 4A,B) and leads to pSrc co-localization at podosomes (Fig. 4C). This data supports data from others [28] demonstrating podosome formation (whether due to physical force or chemical induction) is mediated by Src.

### Podosome formation in response to physical cues is mediated by Src kinase

**Fig 5:**
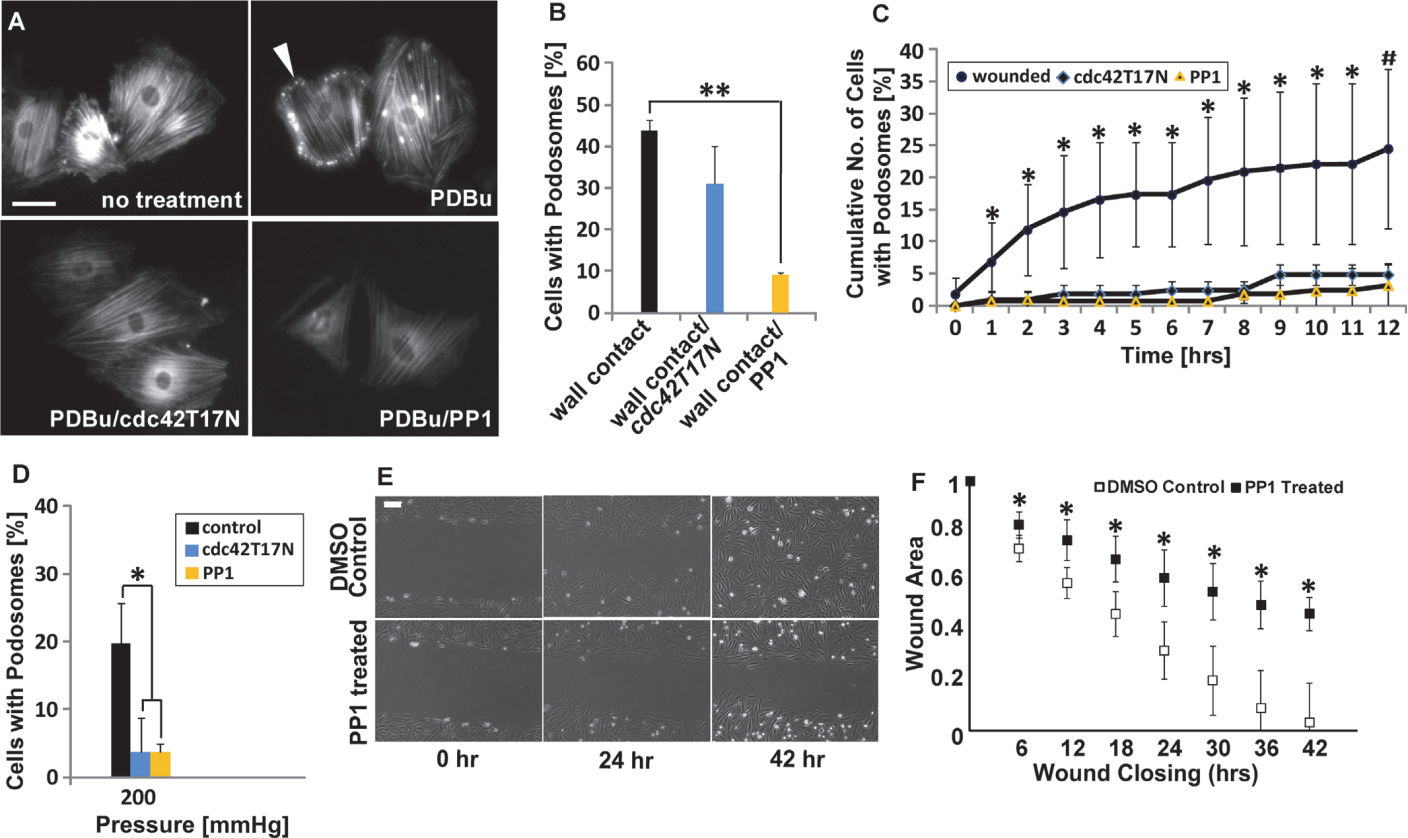
Podosome formation induced by physical barriers, wounding and hypertensive pressure are dependent on Src activity. (A) Representative images of A7R5 cells treated with PDBu with or without 10 μM PP1 or cdc42T17N pretreatment. White arrow indicates podosome location. Scale bar, 50 μm. (B–D). Percentage of cells in exhibiting podosomes with or without PP1 or cdc42T17N pretreatment when in contact with a vertical wall (B), when wounded (C), or when stimulated with increased pressure of 200mmHg (D). Data are mean ± S.D.* P < 0.05, **P<0.01, (Student’s *t*-test, n = 3), # P<0.05 (Wilcoxon rank sum test for wounded compared to PP1 and Student’s *t*-test for wounded compared to cdc42T17N, n = 3). (E) VSMCs with/without PP1 treatment at 0, 24, 42 hours after wounding. Scale bar, 100 μm. (F) Wound closure rate comparison between VSMCs with/without PP1 treatment. Data are mean ± S.D. Data were normalized to 0 hour values, * P<0.05 (Wilcoxon rank sum test, n = 15).

To corroborate the mechanism by which physical cues stimulate podosome formation, we further focused on the Src pathway. Src has been shown to activate cdc42, a Rho family GTPase required for podosome assembly [28]. As such, we investigated Src and cdc42 signaling as possible mechanisms through which physical cues activate podosome formation. To inhibit Src, PP1, a selective and potent Src-family tyrosine kinase inhibitor [29] was used, and to inhibit cdc42 activity, transfection with a dominant negative cdc42 (cdc42T17N) was used. To confirm the effectiveness of PP1 and cdc42T17N in inhibiting podosomes, VSMC were treated with either PP1 or were transfected with cdc42T17N and then stimulated with phorbol 12,13 dibutyrate (PDBu), a known inducer of podosome formation through the Src pathway [6] as a positive control (Fig. 5A). As expected, both PP1 and dominant-negative cdc42 inhibit podosome formation occurring in response to PDBu. Cells were then treated with either PP1 or dominant-negative cdc42 and exposed the cells to either topographical cues (Fig. 5B), wounding (Fig. 5C) or pressure (Fig. 5D). Notably, PP1 inhibits podosome formation in response to topographical cues (Fig. 5B), wounding (Fig. 5C) and 200 mmHg pressure, lowering it to levels of ambient pressure (Fig. 5D). Likewise, cells transfected with cdc42T17N formed fewer podosomes in response to wounding (Fig. 5C) and static pressure (Fig. 5D). Treatment with PP1 also correlates with impaired wound healing (Fig. 5E,F). Cdc42T17N transfected cells in contact with micropost wall also displayed decreased podosome formation, although this decrease was not statistically significant (Fig. 5B). Together, these data suggest that podosome formation in response to physical cues occurs through the Src pathway.

## Discussion

While numerous studies have investigated podosome formation and function, most have induced their formation using phorbol esters such as PDBu as agonists [6]. However, physiological, physical inducers of podosomes have generally been overlooked. The physical cues described in this work are designed to mimic the physical cues introduced pathologically during surgical interventions such as balloon angioplasty or implantation of a graft or stent [21, 30], where the compressive forces exerted on the vessel wall and the topography of tissue-engineered constructs may modulate VSMC migration and matrix remodeling, in part, through podosome formation. These results are critical to our understanding of the factors regulating podosomes and their potential role in cardiovascular disease.

Abnormal vascular smooth muscle cell migration and intimal hyperplasia occur as responses to vascular injury and with the progression of atherosclerosis. Quiescent VSMCs become migratory, moving from the medial layer into the intima and depositing extracellular matrix resulting in intimal thickening. Podosomes have been shown to actively degrade matrix *in vitro* and are hypothesized to aid in VSMC migration across tissue boundaries [1]. Recently, Quintavalle and co-workers observed podosome formation *ex vivo* in aortas of miRNA-143(145) knockout mice [7], further implicating podosomes as possible contributors to VSMC migration. Together with these studies, our results point to a potentially important role for podosomes in VSMC invasion during diseases such as atherosclerosis or vascular injury. Our *in vitro* results suggest that direct tissue damage resulting from surgical intervention may activate podosome formation thus causing aberrant VSMC migration, and raise the intriguing possibility that podosomes may be potential therapeutic targets.

Vascular injury, like that which occurs during angioplasty or vein graft implantation [9], also induces VSMC migration *in vivo*. The topographical landscape of the cellular microenvironment is known to alter cell migration due to the contact guidance cues it provides. Contact guidance cues can change a cell’s proliferation, migration, or direction of movement [11]. To mimic this injury and topographical cues present in the healing vascular microenvironment, we used a scratch assay and micromolded substrates. Wounding leads to the formation of podosomes at the leading edge of cells. Our data also indicate that contact guidance cues through either the presence of topographically patterned walls or wounding trigger podosome formation. Since contact guidance is known to encourage persistent migration, our results suggest that podosome formation may be one facet of directional migration.

Hypertensive pressure often occurs with atherosclerosis and is known to have deleterious effects on endothelial and vascular smooth muscle cell function [31]. Since previous studies show pulsatile [32,33] and static pressures [13] increase VSMC migration, we investigated pressure as a potential stimulator of VSMC podosome formation. Applied pressure of 200 mmHg, which mimics pressures found in stage II hypertension, significantly increased podosome formation compared to normotensive (100 mmHg) and stage I hypertensive (150 mmHg) pressures (Fig. 3B). These results are complementary to recent findings by Aga *et al*., who determined that trabecular meshwork cells may respond to increased intraocular pressure by forming podosomes capable of releasing MMPs and degrading their surrounding ECM [34]. Based on these findings, it is possible that VSMCs experiencing hypertensive pressures assemble podosomes to aid in their migration, contributing to disease progression. Future studies should investigate podosome formation *in vivo* in a hypertensive model.

Src is known to stimulate podosome formation as well as be involved in podosome disassembly in a variety of cells and systems [2]. Importantly, our data indicate that Src regulates the formation of podosomes in response to the physical cues described here, suggesting that similar molecular mechanisms are at play. Src is a component in a miRNA143/145 pathway that regulates podosome formation [7]. The cdc42 GTPase is also involved in podosome maturation [2]. Cdc42 is involved in the assembly process of podosomes, where cortactin, N-WASP, and the Arp2/3 complex form a larger complex with cdc42, WASP and dynamin [35]. Podosome formation in response to topography, wounding and hypertension studies was inhibited by a Src inhibitor or dominant negative cdc42 (Fig. 4). These data suggest that topography, wounding, and increased pressure upregulate Src and cdc42 activity, increasing podosome formation. However, how this upregulation occurs in response to the physical stimuli remains unknown and is an area of interest requires further experimentation. Also, as cdc42N17 did not completely inhibit podosome formation within cells contacting walls, it is likely parallel pathways exist leading to podosome formation as well [28].

Podosomes have been shown to form in a number of cell types including osteoclasts, macrophages, endothelial cells, and dendritic cells [4,5,36]. It remains unclear if the same physical cues identified here will also trigger podosomes in other cell types as well, which is of future interest. Additionally, it would be of significant interest to recapitulate these results *in vivo* or in human primary cells. Moreover, there are many parallels between podosomes and invadopodia, actin-based cellular structures present in cancer cells that are thought to enable metastasis by releasing MMPs to facilitate invasion [35]. Podosomes and invadopodia contain many of the same intracellular proteins and have matrix degrading ability, however podosomes are typically more transient, forming and receding in a matter of minutes, where invadopodia persist for longer periods [37,38]. It has been suggested that podosomes may be precurors to invadopodia [35]. As such, it is of future interest to determine if the same physical cues that trigger podosomes in VSMCs also trigger invadopodia in cancer cells, since many of these same cues exist in the tumor microenvironment.

### Conclusion

Our study identifies three physical cues—topography, pressure, and wounding—as novel, physiologically-relevant stimulators of podosome formation. Since podosomes form in many other cells types, it is possible that these same physical cues which induce podosome formation here may also cause podosome formation in a number of pathologies.
